# Alzheimer’s 2030: From Precision Genomics to Artificial Intelligence

**DOI:** 10.3390/genes17020233

**Published:** 2026-02-12

**Authors:** Valeria D’Argenio, Rossella Tomaiuolo, Silvia Bargeri, Giulia Sancesario

**Affiliations:** 1 Department of Human Sciences and Quality of Life Promotion, San Raffaele Open University, Via di Val Cannuta 247, 00166 Rome, Italy; dargenio@ceinge.unina.it; 2 CEINGE-Biotecnologie Avanzate Franco Salvatore, Via G. Salvatore 486, 80145 Naples, Italy; 3 Università Vita-Salute San Raffaele, 20123 Milan, Italy; 4 IRCCS Galeazzi Sant’Ambrogio, 20157 Milan, Italy; 5 Unit of Clinical Epidemiology, IRCCS Istituto Ortopedico Galeazzi, 20157 Milan, Italy; silvia.bargeri@grupposandonato.it; 6 Biobank and Clinical Neurochemistry, IRCCS Santa Lucia Foundation, 00179 Rome, Italy; g.sancesario@hsantalucia.it; 7 European Center for Brain Research, 00143 Rome, Italy

**Keywords:** Alzheimer’s Disease, neurogenomics, gender, artificial intelligence, digital health, GWAS, data treatment, ethics

## Abstract

Alzheimer’s disease (AD) represents a critical global health challenge, with its prevalence and associated costs expected to double significantly by 2030 and 2050. While lifestyle interventions are crucial, sporadic late-onset AD has a substantial genetic component (40–80% heritability), though known variants limit the scope of traditional precision medicine. Crucially, sex and gender are significant risk determinants, with women accounting for two-thirds of cases due to a complex interplay of biological and sociocultural factors. This review focuses on the influence of genetic and gender-related factors, examining large-scale genome-wide association studies (GWASs) and their role in developing advanced genetic risk scores (GRS) for precision genomics. We also explore the potential of Artificial Intelligence (AI) for multimodal big data analysis and digital health tools to promote personalized prevention and emerging concerns about ethics, privacy and data treatment. The convergence of these findings underscores the urgent need for a genetic-, sex- and gender-informed precision-medicine approach to AD.

## 1. Alzheimer’s 2030

When it was published 10 years ago, the 2015 World Alzheimer’s Report was the first to highlight the dramatic increase in the global burden of dementia, with more than 50 million people worldwide expected to be living with dementia by 2020. This number was estimated to almost double every 20 years, reaching 82 million in 2030 and 152 million in 2050. At the same time, the annual global cost of dementia of above US$ 1.3 trillion was expected to rise to US$ 2.8 trillion by 2030. Great efforts have been made to contain this growth, undoubtedly associated with the increase in the aging and life expectancy of the general population, both in industrialized and emerging countries. The 2023 Report focused on strategies to reduce the incidence by identifying modifiable and non-modifiable risk factors and promoting lifestyle changes, e.g., a healthy diet, no smoking, and a non-sedentary lifestyle. However, evidence suggests that sporadic late-onset Alzheimer’s Disease (AD), the most frequent form of dementia, has a substantial genetic component, with heritability estimated to be between 40 and 80% [1,2]. So, is it possible to counterbalance the interactions among genes, environmental, and lifestyle factors that interfere with the onset and development of this inexorable disease? Today, advances in genomics have highlighted the importance of certain variants in the development of AD, although known associated single-nucleotide polymorphisms (SNPs) can explain about 30% of cases, with the majority of risk attributed to variants within APOE [3]. This essentially limits the aim of precision medicine, already widely used in the treatment of tumors, of stratifying patients based on their genetic-biochemical profile in order to apply the most effective therapy [4]. Among the multiple axes of variability, sex and gender have emerged as significant determinants of AD risk and phenotype. Women account for nearly two-thirds of all AD cases. Substantial evidence now indicates that this difference cannot be attributed solely to longer female life-expectancy [5,6]. Instead, a complex interplay of biological, genetic, hormonal, immune, and sociocultural factors shape sex- and gender-specific trajectories across the AD continuum. For this reason, current research exploits extensive, multi-center and transnational collections of clinical, genomic, biochemical, imaging, etc., data built up over the last few decades, in which the powerful potential of artificial intelligence (AI) can be applied.

Although AD shares several pathogenic features with other neurodegenerative disorders, including Parkinson’s disease (PD) (such as age-related vulnerability, neuroinflammatory processes, mitochondrial dysfunction, and polygenic contributions), important disease-specific distinctions justify a focused investigation of AD. In particular, AD is characterized by the central and early involvement of amyloid-β and tau pathology, a uniquely strong genetic influence of APOE, and a highly polygenic risk architecture that differs in scale and structure from that observed in PD and related conditions. Moreover, the very long AD preclinical phase, together with the availability of validated fluid and imaging biomarkers, makes it uniquely suited for precision genomics approaches aimed at early risk stratification and prevention. While cross-disease comparisons can provide valuable insights into shared neurodegenerative mechanisms, delineating AD-specific genomic and biological features remains essential to advance targeted prevention strategies and personalized interventions.

In this review, we focus on cutting-edge research into the influence of genetic and gender-related factors on AD risk. We specifically examine the findings of large-scale genome-wide association studies (GWASs), such as those conducted by the International Genomics of Alzheimer’s Project (IGAP) on multicenter databases. These studies are instrumental in developing advanced genetic risk scores (GRS) for AD, a key component of precision genomics, which are designed to improve the identification of individuals with high AD susceptibility, ultimately enabling more targeted prevention strategies. Furthermore, we explore the potential of integrating AI into AD prevention, both for multimodal analysis of big data from diverse sources and for digital health tools that promote lifestyle changes. A discussion on significant ethical challenges, particularly regarding data privacy related to the use of AI is also present. Taken together, the convergence of biological, epidemiological, and sociocultural findings underscores the need for a sex- and gender-informed precision-medicine approach to AD. Such an approach requires systematic integration of sex as a biological variable, gender as a sociocultural determinant, and gender-bias frameworks as tools to identify and correct structural inequities across the care continuum. This recent evidence examines the methodological challenges that have hindered progress in this area and outlines implications for diagnosis, biomarker development, clinical trial design, and targeted interventions.

## 2. Materials and Methods

This is a narrative review based on a critical and selective synthesis of the scientific literature on genetic, sex-related, and gender-related determinants of AD. Sources were identified through non-systematic searches of PubMed between 2016 and 2025, with particular attention to large-scale GWAS consortia, biomarker studies, AI and sex/gender-focused research published within the selected time window. Additional references were included through backward and forward citation tracking. Given the interdisciplinary scope and the rapidly evolving nature of the fields addressed (genomics, sex and gender medicine, and AI), this review was conceived as a narrative synthesis rather than a systematic review. Consequently, no formal PRISMA protocol or predefined inclusion/exclusion criteria were applied, which may introduce selection bias. To mitigate this limitation, the literature identification combined targeted PubMed searches within a defined temporal window (2016–2025) with backward and forward citation tracking of key articles from large consortia and high-impact studies.

## 3. Results

The literature review highlighted three complementary thematic areas—precision genomics, sex and gender determinants, and applications of AI—that serve as interpretative cornerstones for discussing the most recent findings in AD.

### 3.1. AD and Precision Neurogenomics

AD represents a paradigmatic example of how precision genomics can reshape the understanding of complex neurodegenerative disorders. The advent of next-generation sequencing and advanced omics platforms has transformed our ability to dissect its molecular basis, enabling a shift from single-gene to genome-wide investigations [7]. Indeed, while less than 5% of cases are explained by highly penetrant mutations in *APP*, *PSEN1*, and *PSEN2* genes [8], large GWASs have demonstrated that late-onset AD (LOAD) is driven by the cumulative effect of numerous common and low-frequency variants, which, if individually considered, have a modest effect size [9,10]. As a consequence, extremely large sample sizes are required to achieve genome-wide significance and to capture the full extent of the polygenic risk architecture underlying the disease.

#### 3.1.1. AD and the Polygenic Risk

In this context, the International Genomics of Alzheimer’s Project (IGAP) was specifically established to increase the statistical power for locus discovery by aggregating GWAS data across multiple consortia and creating publicly accessible summary statistics for downstream analyses. In its first two-stage meta-analysis, carried out on 4 previously published GWAS data sets, 74,046 individuals of European ancestry (17,008 cases and 37,154 controls in stage 1, followed by independent replication) were totally analyzed [11]. By imputing more than 7 million SNPs, in addition to confirming the *APOE* and other already known loci, it was possible to identify 11 novel susceptibility loci, highlighting pathways related to lipid metabolism, endocytosis, and innate immunity [11].

Subsequent IGAP-extended meta-analyses further expanded the number of AD-related significant loci by identifying other 5 new loci (*QCK*, *ACE*, *ADAM10*, *ADAMTS1*, and *WWOX*), an immune-mediated disease haplotype (HLA-DR15) within the HLA locus, and collectively implicating >70 loci involved in amyloid, tau, immune, and microglial pathways [12].

A further milestone in AD genomics was reached with the large, multi-ancestry GWAS published by Bellenguez and colleagues in 2022, which expanded both sample size and population diversity beyond those of previous IGAP-driven studies [13]. By combining more than 111,000 clinically diagnosed AD cases and over 677,000 controls from European and non-European cohorts, this study not only confirmed well-established risk loci (such as *APOE*, *BIN1*, *TREM2*, and *PICALM*) but also identified 42 additional genome-wide significant regions, increasing the total number of AD-associated loci to more than 70. Many of these newly discovered loci were strongly enriched for genes involved in microglial activation, synaptic regulation, and endosomal trafficking, such as *LILRA5* and *ITGAM*, thereby reinforcing the central roles of innate immune pathways and lipid metabolism in AD pathogenesis. Importantly, this work demonstrated that several variants exhibit substantial differences in allele frequency and effect size across ancestries, providing some of the first robust evidence that heterogeneity in genetic risk for AD is not uniformly distributed across populations. Moreover, the study showed that integrating multi-ancestry data significantly improves the performance and portability of polygenic risk prediction, highlighting the need to broaden discovery efforts beyond populations of European descent. Collectively, these findings indicate that expanding GWAS to diverse ancestries is essential not only to increase the number of risk loci but also to enhance the accuracy of precision genomics in AD across global populations.

These large-scale GWASs have not only broadened our understanding of the LOAD molecular bases, but have also provided the statistical foundation for polygenic risk models. As risk alleles accumulate across hundreds of loci with small to moderate effects, polygenic risk scores (PRSs) have emerged as powerful tools to quantify individual genetic liability [14]. In this manuscript, we use PRS as an umbrella term for genome-wide polygenic scores derived from GWAS summary statistics across many loci, whereas we reserve the term GRS for more restricted scores built from a limited set of established risk variants (typically genome-wide significant loci or pathway-/mechanism-focused panels). Accordingly, GRS is used when referring to parsimonious, locus-selected scores, while PRS is used for broader, genome-wide models. This aspect is crucial for stratifying asymptomatic individuals, refining risk prediction across ancestries, and potentially guiding targeted prevention strategies. Methodological refinements, including pruning-and-thresholding, linkage disequilibrium-aware Bayesian approaches, and penalized regression, have steadily improved prediction performance, with AD PRS models now achieving area-under-the-curve (AUC) values around 0.70–0.75 when combined with age and APOE, and identifying individuals in the top few percentiles of the PRS distribution who have a three- to five-fold increased risk of AD compared with the population average [15].

In particular, a key step toward translating GWAS discoveries into individual-level prediction was taken by Zhang and colleagues, who investigated the discriminative ability of polygenic risk models to accurately identify LOAD genetic risk [16]. By leveraging large-scale GWAS summary statistics and testing multiple modelling strategies across independent cohorts, the authors demonstrated that risk prediction for AD is best explained by a limited number of loci with relatively larger effects, rather than by a fully diffuse genomic architecture. This observation supports an “oligogenic-like” pattern, in which a substantial portion of the predictive power arises from a restricted set of common and low-frequency variants, many of which are located in or near genes involved in lipid metabolism, endosomal processing, and microglial activation, alongside the dominant influence of APOE ε4. Notably, their models revealed that incorporating thousands of additional variants with extremely small effects marginally increases predictive accuracy, whereas weighting variants by functional category or linkage disequilibrium structure substantially improves model performance. These findings underline that effective polygenic risk estimation for AD does not rely on indiscriminate inclusion of all variants across the genome, but on the careful integration of disease-relevant loci, thereby providing further support for the development of biologically informed PRS that reflect the underlying pathways driving AD susceptibility [16].

More recent work extends this framework by deriving cell-type-specific or pathway-focused PRS (e.g., microglia- or synapse-weighted scores) and integrating PRS with plasma and cerebrospinal fluid biomarkers, neuroimaging, and digital cognitive measures to improve preclinical risk stratification and to dissect the relative contribution of distinct biological processes to disease onset and progression.

In particular, O’Neill and colleagues developed a novel multi-omic framework to generate cell-type-specific PRS (ct-PRS) for AD by integrating single-nucleus RNA-seq and ATAC-seq chromatin accessibility datasets with large GWAS summary statistics [17]. These ct-PRS models partition the genome by regulatory activity across discrete neural and glial cell populations, then aggregate variant weights within each partition to estimate individual genetic liability associated with specific cell-type regulatory programs. In their analyses, they found that ct-PRS derived from microglia- and astrocyte-specific regulatory landscapes were differentially associated with key AD endophenotypes: for example, the astrocyte-ct-PRS showed stronger links to amyloid-β plaque burden, while the microglia-ct-PRS was more strongly associated with tau pathology, activated microglia markers and cognitive decline. Importantly, models that emphasized variants located in cell-type-specific accessible chromatin regions (snATAC-seq) exhibited greater cell-type specificity (lower inter-score correlation) compared to those derived from transcriptomic specificity alone, indicating the value of functional regulatory annotation in refining risk stratification. By showing that distinct cell-type modules of genetic risk map to discrete pathobiological processes and disease stages, this work provides a compelling demonstration of how precision genomics can move beyond aggregated genome-wide risk and instead align genetic liability with the underlying cellular biology of AD [17].

Aligning with this multi-omic perspective, Venkatesh and colleagues recently demonstrated that integrating genomic, transcriptomic, and epigenomic data can substantially refine both molecular interpretation and risk prediction for AD [18]. By combining GWAS-derived variants with gene expression and regulatory annotations from disease-relevant brain tissues, the authors identified convergent pathways centered on neuroinflammation, lipid metabolism, and synaptic dysfunction, many of which were driven by variants acting within microglial and astrocytic regulatory networks. Importantly, they showed that predictive models incorporating multi-omic features outperform traditional genome-wide PRS, revealing that functional prioritization of variants within biologically active regions increases accuracy across both case–control classification and quantitative biomarker traits. These findings reinforce the notion that effective genetic risk estimation in AD is enhanced not by indiscriminate accumulation of genome-wide signals, but by weighting variants according to their mechanistic relevance, ultimately moving polygenic prediction toward biologically informed and clinically applicable precision genomics [18].

Together, these emerging frameworks indicate that the future of AD polygenic modelling will rely not only on larger datasets but also on biologically informed and machine-learning (ML)-driven strategies that enhance interpretability and clinical applicability.

#### 3.1.2. AI Tools for Precision Neurogenomics in AD

A novel methodological development in the direction of AD polygenic modelling is represented by BrainGeneBot, a transductive learning framework proposed by Qu and colleagues to enhance variant prioritization and biological interpretability in PRS studies by leveraging large language models [19]. BrainGeneBot integrates functional annotations, gene regulatory networks and multi-tissue expression profiles with a generative pretrained transformer model specifically adapted for brain-focused genomics. Rather than indiscriminately weighting variants according to statistical effect size alone, the system generates biologically informed embeddings that prioritize variants based on predicted molecular impact, cell-type specificity, and pathway relevance. Importantly, the framework demonstrated improved identification of mechanistically meaningful variants within immune-microglial, synaptic and lipid-processing pathways, and increased the biological interpretability of PRS models without sacrificing predictive performance. By combining machine learning-based generative models with multi-layer functional annotation, BrainGeneBot exemplifies a new direction in precision genomics, in which risk prediction tools are explicitly designed to reflect the molecular complexity of AD, moving PRS development beyond traditional statistical aggregation and toward transformer-informed, mechanistically interpretable architectures.

Despite these achievements, current AD PRS models exhibit reduced portability across ancestries because most GWAS, including IGAP, have been conducted primarily in populations of European descent, necessitating efforts to build cross-ancestry PRS and to generate adequately powered GWAS in non-European populations [20,21]. In particular, Nicolas and colleagues have recently addressed a key limitation in the clinical applicability of AD polygenic risk models by evaluating the transferability of European-derived PRS across diverse ancestral groups [20]. By testing widely used PRS algorithms in cohorts of African, Asian, Latino, and mixed ancestry, they demonstrated a significant reduction in predictive performance when models were trained exclusively on European GWAS datasets, with effect sizes dropping by more than half in some non-European populations. This reduced portability was primarily attributed to differences in allele frequencies, linkage disequilibrium structure, and ancestry-specific regulatory architectures, which alter the relative contribution of multiple loci, including APOE ε4. Importantly, the study also showed that even modest increases in ancestry diversity within discovery GWAS substantially improve cross-population prediction, and that incorporating ancestry-matched linkage disequilibrium references or functional annotations yields further gains. These findings emphasize that precision genomics in AD cannot be globally implemented without broadening multi-ancestry discovery efforts and adapting PRS development to population-specific genetic landscapes, thereby ensuring that genomics-based risk stratification evolves equitably alongside advances in prediction accuracy. From a quantitative standpoint, current AD PRS models typically achieve area-under-the-curve (AUC) values in the range of ~0.70–0.75 when combined with age and APOE genotype in populations of European ancestry, whereas performance is consistently reduced in non-European cohorts, with reported AUC values often declining to ~0.55–0.65 depending on ancestry and study design [15,16,20]. Notably, recent multi-ancestry analyses have shown that even modest increases in ancestral diversity within discovery GWAS datasets can partially mitigate this performance gap, underscoring the importance of inclusive genomic resources for clinically meaningful risk prediction [20].

These trends align with emerging diagnostic technologies in AD, in which the multimodal integration of imaging, genomic, and proteomic biomarkers, combined with non-invasive platforms, offers complementary avenues for early detection and risk stratification [22]. This perspective resonates with broader precision medicine frameworks, which propose that genomics-guided risk stratification should be integrated with multimodal biomarkers and tailored therapeutic strategies to achieve patient-specific disease modification in AD [23].

All the findings discussed in this section are summarized in Table 1.

Taken together, these data suggest that polygenic risk modelling is now poised to move beyond discovery research and into clinically meaningful applications, where equitable, biomarker-integrated and ancestry-aware PRS may support the early identification of at-risk individuals and guide precision prevention strategies in AD. Within a precision medicine paradigm, AD genomics is therefore evolving from single-gene and APOE-centric approaches toward multidimensional, polygenic and multi-omic models, enabling genetically informed trial enrichment, risk-adapted prevention, and, potentially, the tailoring of disease-modifying therapies to specific molecularly defined subgroups.

However, despite their growing methodological sophistication, current PRS models for AD face significant limitations that constrain their clinical transferability. Predictive performance remains modest at the individual level, even when PRSs are combined with age and APOE genotype, and their added value beyond established risk factors is still debated. Moreover, most PRSs have been derived from predominantly European-ancestry cohorts, resulting in reduced portability and potential misclassification in underrepresented populations. These limitations raise concerns about premature clinical implementation and underscore the need for ancestry-aware discovery efforts, careful calibration, and the integration of PRSs within multimodal risk models, rather than their use as standalone predictive tools.

### 3.2. Sex and Gender Differences in AD

For an accurate interpretation of the evidence reported in this session, it is essential to distinguish, from the outset, between biological sex and gender, as these terms are often used in overlapping ways [24]. Biological sex encompasses genetically and physiologically determined factors [25].

In contrast, sociocultural gender reflects environmental and structural determinants such as educational and occupational opportunities, caregiving roles, chronic stress exposures, health behaviors, diagnostic norms, and broader cultural expectations that shape cognitive reserve, symptom recognition, and access to care [25].

Moreover, many clinical manifestations and disease-related biomarker differences cannot be attributed exclusively to intrinsic biological vulnerability or to gendered life-course exposures; instead, they emerge from their reciprocal reinforcement. These sex- and gender-related interactions shape diagnostic timing, pathological burden, and treatment response, revealing patterns that become visible only when both dimensions are considered jointly [26]

These three conceptual distinctions underpin the organisation and interpretation of the subsequent sections. A synthesis of the major biological and sociocultural determinants, together with their interactions and alignment with current evidence, is presented in Table 2.

#### 3.2.1. Sex-Based Biological Determinants of AD

Epidemiological studies consistently show that women account for the majority of AD dementia cases [5,6], a pattern confirmed in large clinical cohorts [48]. Although incidence patterns vary across studies, biological factors explain substantial sex differences in disease vulnerability and expression [5,6].

Biological mechanisms, such as hormonal changes, play a central role: estrogen exerts neuroprotective effects on synaptic plasticity, mitochondrial function, and amyloid-tau homeostasis; its abrupt decline during menopause accelerates brain aging and increases susceptibility to metabolic and inflammatory dysregulation related to AD [28], as well as other diseases of aging, such as PD [49,50]. Conversely, testosterone may confer neuroprotection in men, although APOE-ε4 may attenuate androgen receptor signaling and its immunomodulatory effects [27,29].

Genetics align with these hormonal patterns, showing sex-specific effects. APOE-ε4 confers a greater relative risk in women, with stronger associations between ε4, phosphorylated tau, and cognitive decline [29,32]. Transcriptomic analyses reveal broader dysregulation of immune, synaptic, and lipid metabolism pathways in female AD brains with comparable APOE burden [33].

In addition, neuroinflammatory processes exhibit pronounced sexual dimorphism: women show higher microglial activation thresholds, region-specific activation patterns, and stronger coupling of innate immune markers (i.e., sTREM2 and clusterin) with alterations in tau and neurofilament light [31,35].

Additional divergence emerges in lipid metabolism, as shown in a recent lipidomic study, which reported that women with AD show reduced levels of unsaturated lipids and omega-3-bearing species [51], a vulnerability not consistently observed in men, suggesting sex-specific interactions among APOE genotype, membrane lipid composition, and neuronal homeostasis [37,38]

Biomarker studies confirm more severe tau accumulation in women at similar amyloid levels, with stronger amyloid-tau coupling, strengthening the notion of sex-specific disease susceptibility [39,51]. This pattern is reinforced by recent multimodal evidence: in a recent multicenter study, women showed higher tau-Positron Emission Tomography (PET) signal and elevated plasma Glial Fibrillary Acidic Protein (GFAP), together with a stronger amyloid/tau association, whereas men displayed stronger tau/cognition and tau/neurodegeneration associations [35].

Despite this robust evidence of biological differences, sex remains insufficiently captured across the AD therapeutic pipeline. In fact, women remain underrepresented relative to their disease burden. They are often not analysed using sex-stratified methods, limiting the detection of sex-specific efficacy and safety signals [43,44]. In parallel, observational and mechanistic work on anti-amyloid therapies has highlighted that the risk of amyloid-related imaging abnormalities (ARIA) is modulated by APOE-ε4 status, baseline amyloid/tau burden, and cerebrovascular pathology, with sex emerging as a relevant, though incompletely characterized, risk factor in multivariable models [41,42,52].

#### 3.2.2. Lifetime Gender Exposures and Sociocultural Determinants

Lifetime gender exposures shape the risk, resilience, and clinical presentation of AD regardless of biological sex. Global analyses demonstrate that sex/gender patterns in AD vary in parallel with regional disparities in education, occupational opportunities, socioeconomic status, and access to healthcare [6]. Women born before the 1960s often had limited educational attainment and lower occupational complexity, resulting in reduced cognitive reserve. Although women may show greater cognitive compensation in early life, they experience a steeper decline as neuropathology accumulates [30].

Gendered caregiving responsibilities, disproportionately borne by women, contribute to chronic psychosocial stress, depression, and sleep disruption, all established risk factors for dementia [34]. Gender norms influence health behaviors, adherence to preventive strategies, and access to medical care. Structural barriers can delay diagnosis in women, compounded by the diagnostic bias of cognitive tests focused on verbal memory, which underestimate early impairment due to women’s baseline performance advantage [40]. Men and women also exhibit distinct clinical phenotypes influenced by gendered behavioral patterns and social expectations: women more frequently exhibit episodic memory deficits, while men exhibit more visuospatial impairment and behavioral symptoms such as apathy or disinhibition [53]. Finally, gender shapes participation in clinical research. Women are underrepresented in studies of disease burden, and sex/gender-stratified analyses are rarely prespecified, limiting the detection of gender-related differences in treatment access, safety, or efficacy [43,44].

#### 3.2.3. Interactions Between Sexual Biology and Gendered Environments

Sexual biology and gendered environments are not independent determinants; their mutual interaction generates emergent phenomena that shape AD risk, clinical trajectories, and pathology.

An explicit intersectional perspective further refines the interpretation of sex and gender differences in AD [6]. Socioeconomic status (SES), encompassing education, occupational complexity, income, and access to healthcare, acts as a key modifier of sex-specific risk across diverse populations. Women with lower educational attainment or limited occupational opportunities often exhibit reduced cognitive reserve, which amplifies the impact of biological vulnerabilities. Conversely, in higher-SES contexts, greater cognitive reserve may delay clinical manifestation despite comparable neuropathological burden, particularly in women.

Importantly, SES-related disparities also influence cardiovascular risk profiles, healthcare access, and diagnostic timing, thereby modulating sex-specific disease trajectories [6]. In low- and middle-income settings, gendered inequities in education and health-seeking behavior may exacerbate late diagnosis in women, while in men, socioeconomic disadvantage is more frequently associated with higher vascular comorbidity and mixed dementia phenotypes.

Hormonal transitions across the female lifespan (e.g., menopause) intersect with gendered exposures such as caregiving-related stress, socioeconomic disadvantage, or limited access to preventive care, amplifying metabolic and inflammatory vulnerability [30,34]. Similarly, the effects of APOE-ε4 on amyloid-tau coupling are modified by both biological sex and gender-influenced cardiovascular and lifestyle factors [27]. Microglial activation states reflect the combined effects of biological and environmental influences [34,36].

Female-specific immune trajectories interact with gendered stressors, inflammatory burden, and lifestyle characteristics, strengthening sex-dependent neuroinflammatory responses. Lipid metabolism, already modulated by the APOE genotype and sex hormones, is further shaped by gendered dietary patterns, socioeconomic constraints, and regional disparities in nutritional access. These combined effects contribute to sex-specific biomarker trajectories and differential vulnerability to neurodegeneration [31,35].

Cognitive trajectories also emerge from sex-gender interactions. Women’s verbal memory advantage (biological and sociocultural upbringing patterns) delays the detection of early impairment; once the disease exceeds compensatory thresholds, decline accelerates [40]. Similarly, gendered occupational roles modulate the impact of sex-dependent disease on cognitive reserve [54].

Therapeutic response reflects this multidimensional interaction. Anti-amyloid therapies exhibit risk profiles shaped by sexual biology (vascular integrity, immune reactivity) and gendered exposures (prevalence of hypertension, patterns of comorbidities, age at treatment initiation) [55]. Lifestyle interventions—physical activity, diet, and vascular risk reduction—exert sex-dependent biological effects mediated by gendered behavioral patterns [36,47].

In summary, AD expression results from the dynamic interaction between sex-specific biology and gendered life-course environments: incorporating sex and gender as core stratification variables, rather than post hoc covariates, will be essential to move from average treatment effects to genuinely personalized therapeutic strategies [56].

These observations underscore that sex-related biological risk is not uniform across populations but is dynamically shaped by socioeconomic and structural determinants, reinforcing the need for intersectional frameworks in both research and clinical practice, as highlighted by global dementia prevention frameworks and sex- and gender-sensitive epidemiological models [34].

### 3.3. AI Applications in AD Prevention and Digital Health

Research in primary prevention spanning decades has pinpointed various biological, behavioral, environmental, and social factors that either heighten or diminish the risk of dementia. These encompass potentially hundreds or thousands of genetic and non-genetic elements that forecast disease risk and progression [57]; however, most do not show a simple, linear correlation with dementia risk across an individual’s lifetime [34].

A recent Special Issue of *Alzheimer’s & Dementia Journal* offers a thorough overview of current AI use in AD and other related dementias, outlining eight primary areas where AI-driven innovation can accelerate research and knowledge. These include new experimental models [58], drug discovery and trials optimization [59], genetics and omics [60], biomarkers [61], imaging [62], methods optimization [63], up to applied models, digital health [64], and prevention [65]. Here, we focus on the application and expectations of AI in managing modifiable dementia risk factors, assessing personal risk, improving individual lifestyles, and consequently, personalizing interventions and monitoring over time.

#### 3.3.1. AI and Dementia Risk Profile

**Figure 1:**
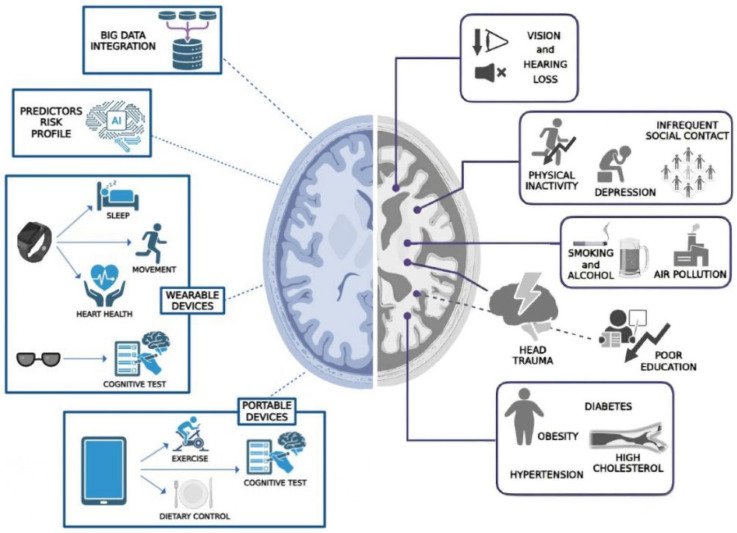
The fourteen modifiable risk factors for dementia across the life course, as identified by the WHO and the most recent Lancet Commission on Dementia [66]. AI-driven strategies for AD prevention are increasingly common, often involving multimodal data analysis and the detection of predictive factors to pinpoint high-risk individuals. Furthermore, the proliferation of portable and wearable technology enables the continuous monitoring of lifestyle behaviors. This sustained measurement offers a better understanding of these habits, thereby promoting modifications to variable risk factors.

According to dementia prevention guidelines from The World Health Organization (WHO) and the most recent Lancet Commission on Dementia report, fourteen modifiable risk factors for dementia have been identified, which point over the course of the human lifespan. These include poor education in early life; hearing and vision loss, excessive alcohol consumption, hypertension, obesity, and high cholesterol, head trauma during mid-life; whereas in later life physical inactivity, smoking, diabetes, depression, infrequent social contact, and air pollution [66] (Figure 1).

Therefore, approximately 45% of AD cases are expected to be preventable through early intervention on these factors, since the risk has been shown to be modifiable regardless of APOE genetic status.

Using multimodal data integration, AI can analyze huge, multiple datasets, including clinical data from electronic health records, genomic, neuroimaging, and lifestyle data from wearable devices, etc., covering different risk-related aspects among those recognized. By integrating “big data” from diverse inputs, AI creates a more comprehensive and precise dementia risk profile, much more accurate than using individual data sources, such as traditional clinical scales or the PRS [67]. Conversely, AI can simplify complex data by breaking down numerous variables into a small set of essential predictors, thereby making the interpretation straightforward for individuals [64]. This capability of AI may allow for the identification of high-risk individuals decades before clinical symptoms become apparent, or the prediction of conversion from mild cognitive impairment (MCI) to dementia [68], using novel and previously unexplored clinical and biological fields (Figure 1). Indeed, a deep learning framework, named Eye-AD, has recently been developed and can analyze retinal images captured either as photographs or via optical computed tomography. By analyzing retinal structural biomarkers, the algorithm can predict the risk of AD over the next 5 years for asymptomatic individuals [69]. These findings highlight the enormous, almost unlimited potential for advancing the diagnosis and management of AD. This includes the opportunity to prevent and modify the clinical course through the early identification of individual dementia risk profiles.

#### 3.3.2. Digital Health and AD Prevention

Once an individual’s specific risk profile has been understood, AI may be exploited to create highly personalized and timely interventions that target the most critical modifiable factors [65]. Rather than representing parallel analytical streams, contemporary AI-based models increasingly build upon PRSs, multi-omics data, and sex- and gender-informed features, allowing heterogeneous dimensions of AD risk to be integrated within unified predictive and preventive frameworks.

Wearable devices and other digital health tools provide objective, large-scale, and incidental measurement of data, such as physical activity and sleep quality, that was previously largely subjective [64].

This results in more detailed information with reduced bias and the potential for larger-scale data collection, and offers a highly promising tool for dementia research, particularly for risk reduction, improved diagnosis, and prognosis. As discussed in the previous paragraph, much of this information relates to lifetime gender exposures or sociocultural determinants, with different impacts on individual risk [36,47] [Table 2]. At this point, appropriate medical or specialized AI supervision from staff experienced in managing patients with mild or at-risk cognitive decline is needed to define personalized lifestyle recommendation plans. For example, a high-risk individual with midlife hypertension due to a genetic predisposition to cardiovascular risk and physical inactivity could be eligible for a targeted, AI-guided program focused on blood pressure management and on the aerobic exercise and cognitive training goals most likely to be effective for that individual. Moreover, another critical issue is the role of nutrition-related factors in predicting dementia incidence, which can be assessed using explainable AI models [70].

The advantage offered by wearable devices and smartphone apps is the creation of a massive number of digital biomarkers, collecting data on physical activity, sleep quality, and even cognitive function through gamified assessments. Using AI algorithms, this stream of digital data can be analyzed to identify subtle changes or deviations from health patterns, identifying clusters associated with a normal or pathological condition [64]. Furthermore, a significant advantage over traditional standard cognitive rehabilitation or occupational therapy programs is represented by the ability to activate just-in-time interventions. Then, AI can trigger timely and automated feedback or “nudges” to support adherence to lifestyle changes, providing active support beyond monitoring [64]. For instance, whether a patient’s activity level decreases, temporarily or for medium-long periods, the AI system could suggest a specific activity, encouraging behavior modification in real time. This could also apply to cognitive training tools, where AI is used to create accessible and scalable tools that can be adapted to the user’s current cognitive state, promoting cognitive engagement, a well-known key protective factor for cognitive decline [71]. In this way, the application of AI in the field of dementia helps shift the focus from late-stage diagnosis to primary and secondary prevention, making risk assessment and reduction interventions highly personalized, objective, and integrated into daily life.

#### 3.3.3. AI Tools Validation

The integration of AI into mobile or wearable devices for various aspects of patient management would offer multiple advantages, including reduced economic costs and time, as well as objective and continuous monitoring of individual habits. However, although numerous AI models are being developed and validated, ML is not yet widely used in clinical practice, and very few, if any, specific, user-facing AI-driven apps for primary dementia prevention have completed rigorous, large-scale randomized controlled trials (RCTs). Likewise, evidence that treating these potential risk factors reduces the risk or progression of dementia remains weak [72]. While many mobile applications are available for dementia care or cognitive training, their utility for preventing or delaying dementia in a real-world setting is still emerging. Currently, a major area of interest is the development of risk models [73]. Many AI models are often prototypes developed by research institutions or mentioned in the context of ongoing clinical trials, rather than widely available commercial apps with validated prevention claims. For example, the Finnish Geriatric Intervention Study to Prevent Cognitive Impairment and Disability (FINGER) is a proof-of-concept RCT that uses a multimodal intervention combining lifestyle and risk-factor interventions. Results suggest that, in individuals at risk of cognitive decline, a combination of exercise, cognitive training, dietary improvements, and vascular risk management has the strongest evidence for improving cognitive and functional outcomes [74]. Currently, another randomised, open-label, blinded endpoint trial, the Mobile Health Intervention for Dementia Prevention through lifestyle Optimisation, is assessing the effectiveness and usefulness of remote coaching for self-managed lifestyle changes aimed at reducing dementia risk factors. The study sets primary outcomes, as composite score comprising blood pressure, non-high-density lipoprotein cholesterol, and body mass index; and other secondary outcomes, including the so-called Cardiovascular Risk Factors, Ageing and Dementia risk score and its components, disability, physical activity, depressive symptoms, cognitive functioning, and daily distance moved, etc. Furthermore, an interesting aim is to evaluate other parameters related to diffusion and implementation of mobile tools, such as acceptability, appropriateness, feasibility, fidelity, costs, and sustainability [75].

Thus, there is a need to translate the high technical performance measures of AI tools into devices with demonstrated real-world practical clinical utility [76]. This significant constraint warrants further examination, as addressing regulatory approval processes, performance benchmarks, bias auditing, and explainability criteria. Recognizing the need for quicker public access to effective, innovative, and lifesaving medical technology, global regulatory agencies have developed accelerated pathways. These frameworks streamline processes while ensuring safety, efficacy, and quality, enabling faster market entry and encouraging innovation, particularly for serious and rare diseases [77]. Examples include the U.S. FDA’s Breakthrough Devices Program for faster review of life-threatening or debilitating disease treatments/diagnoses [78], and the EU’s recent Health Technology Assessment Regulation for expedited, evidence-based decision-making [79].

#### 3.3.4. AI and Ethical Challenges

In the next few years, AI tools will contribute to bringing significant benefits to public health and prevention efforts. Nevertheless, AI raises significant ethical challenges, particularly regarding data privacy related to the use of wearable devices. Continuous monitoring with these devices yields a substantial volume of lifestyle and health data. Consequently, it is crucial that patients, caregivers or normal individuals, are fully informed regarding the treatment protocol, the potential benefit and privacy risks associated with their use and transfer, and the protective measures in place [80]. This level of information is especially critical for patients with permanent or transient cognitive impairment, even mild, due to their inherent frailty and vulnerability [81]. Detailed regulations on the use of data in AI research are needed, in addition to Regulatory frameworks, such as the General Data Protection Regulation (GDPR) in the EU and the Health Insurance Portability and Accountability Act in the United States [82].

Recently, the EU has defined the AI Act, specifically focusing on development and application of AI in public healthcare through a risk-based approach [83], classifying low risk for general well-being advice (low-risk) up to high risk in case of support clinical decision-making, which requires more stringent anonymization and cybersecurity measures [84]. The European Health Data Space (EHDS) Regulation, published in March 2025, established a single framework for the use and exchange of electronic health data (EHD) throughout the EU. This regulation aims to enhance individuals’ access to and control over their personal EHD. Additionally, the EHDS facilitates the secure sharing of specific data and enables the secondary use of health data for research and the development of AI, thereby complementing the GDPR and the AI Act [85].

By promoting public literacy and awareness of the risks of digital technologies, combined with this significant improvement in data policies, it is essential to accelerate the achievement of innovative AI results while still ensuring strong data protection.

## 4. Discussion

The year 2030 is a critical benchmark in global AD and dementia initiatives. Today (2026), the prevalence of dementia worldwide is closely and continuously monitored, along with its social impact assessment, in an effort to contain the inexorable growth of this global emergency. With all the progress in both the science of ageing and AI, we can accurately and precisely identify individuals at high risk of developing AD years before any clinical signs, such as a mild cognitive deficit, become apparent [86,87]. The strategies by the AD International focus on promoting approaches to reduce the risk of dementia through lifestyle changes (e.g., a healthy diet and an active lifestyle, etc. [88]). Furthermore, cognitive and psychosocial rehabilitation, or tele rehabilitation, are emerging as an integral part of dementia care, not simply an adjunct to pharmacological treatments [89]. In this review, we focused on non-modifiable genetic and gender-related factors affecting the risk of AD, and on the potential application of AI to reduce the weight of modifiable factors on risk. Much of the genetically based heritability of AD remains unmapped to causal genetic factors; this so-called “missing heritability” is common to many complex diseases [90] and represents a challenge for precision genomics. However, these shortcomings call for triangulating with other causal modelling methods that incorporate biological, environmental, sociocultural, and gender factors to help us draw more reliable conclusions about whether a risk factor is causal for dementia.

Effective dementia prevention relies on establishing the causal relationship between risk factors and the disease’s development. Therefore, a crucial step in maximizing prevention efforts is to determine whether both modifiable and non-modifiable risk factors are genuinely causal. A key challenge is that observed associations, such as those involving genetic and gender risk factors, might not indicate direct causation but could instead reflect shared causality, confounding variables, or reverse causation. Thus, understanding the true causal roles and trajectories of these risk factors is essential [91].

From an interpretative standpoint, it is important to emphasize that many of the associations discussed between genetic variation, sex, gender-related factors, and AD risk are derived from observational and stratified analyses, and should therefore not be interpreted as inherently causal. While GWAS and sex-aware genetic analyses provide critical insights into risk modulation and biological heterogeneity, they do not, on their own, establish causal relationships. In this context, causal inference frameworks such as Mendelian randomization, triangulation approaches, and genetically informed mediation analyses offer powerful complementary strategies to disentangle causal effects from confounding and reverse causation. The integration of these methodologies into future multi-ancestry and sex-aware genomic studies will be essential to strengthen causal interpretation and to translate precision genomics into robust prevention and intervention strategies.

Recent years’ evidence demonstrates that sex and gender differences in AD are fundamental determinants of disease vulnerability, neuropathological burden, cognitive trajectory, and treatment response. Despite this, the integration of sex- and gender-specific knowledge into routine clinical practice and research design remains limited [92].

Adopting an intersectional lens reveals that sex and gender differences in AD are contingent upon socioeconomic and structural contexts. Failure to account for socioeconomic status risks obscuring meaningful heterogeneity within sex-stratified groups and may limit the generalizability of findings across populations [34].

A major obstacle arises from the gender bias in medicine. Gender bias can emerge when clinicians assume that women and men are essentially the same or should be treated identically, despite meaningful differences in biology, disease patterns, living conditions, and life experiences. It can also emerge when differences are presumed where none actually exist, especially when rigid, dichotomous stereotypes about women and men are taken as legitimate [93].

The first “assuming sameness” view reflects the long-standing practice of applying uniform diagnostic cut-offs for amyloid and tau biomarkers, despite growing evidence that women reach abnormal thresholds at lower levels of cognitive impairment and at lower amyloid burden than men. Neuroimaging and biomarker studies demonstrate that women show earlier and more extensive tau accumulation for a given amount of β-amyloid [39] and exhibit metabolic and neuropathological alterations at milder clinical stages [28].

Similarly, most clinical trials before 2024 reported aggregated results without testing sex-by-treatment interaction effects [5]. These practices obscure sex-specific vulnerability patterns and delay recognition of clinically meaningful divergence in disease progression, especially the steeper post-MCI decline documented in women [54]. From an intersectional perspective, AD clinical research continues to include participants who are not representative of real-world epidemiology, with about 95% of trial participants being white [94]. This narrow evidence base limits our understanding of AD pathogenesis, reduces the generalizability of findings, and reinforces existing health inequities [95,96].

Despite their growing predictive performance, the real-world clinical implementation of PRSs in asymptomatic individuals remains challenging. To date, there are no universally accepted clinical thresholds that define actionable levels of GRS, and PRS values should be interpreted as continuous, probabilistic modifiers rather than binary indicators of disease. Moreover, the clinical meaning of a given PRS percentile varies substantially across ancestries, age groups, and baseline risk profiles, limiting the direct transferability of fixed cut-offs. Ethical considerations, including the potential psychological impact of risk disclosure, the risk of overmedicalization, and issues related to informed consent and data governance, further complicate clinical deployment. For these reasons, current evidence supports the use of PRS primarily as a tool for research stratification and trial enrichment, or as one component of integrated risk models combining genomics, biomarkers, lifestyle factors, and structured counseling frameworks, rather than as a standalone screening test.

To prevent AI-driven precision medicine from exacerbating existing health inequities, sex, gender, and ancestry should be jointly modeled rather than treated as independent or secondary variables. From a methodological perspective, this requires multivariable, interaction-based frameworks that incorporate biological sex, genetic ancestry, and gender-related sociocultural factors simultaneously into risk prediction models. Such approaches allow the estimation of sex-by-ancestry and gender-by-ancestry interaction effects, which are critical for capturing heterogeneity in genetic risk expression, biomarker trajectories, and clinical outcomes across populations. In the context of PRS, this implies moving beyond ancestry-stratified PRS toward integrative models that combine ancestry-aware weighting with sex-specific effect sizes and adjustment for gender-related exposures, such as education, socioeconomic status, caregiving burden, and health-seeking behavior. Hierarchical and mixed-effects models, as well as multi-task and fairness-aware ML architectures, offer practical solutions to jointly account for these dimensions while preserving interpretability and predictive performance.

Importantly, gender should be operationalized through measurable life-course variables rather than assumed as a proxy for sex. Failure to explicitly integrate gendered exposures alongside sex and ancestry risks embedding structural biases into AI systems, thereby reinforcing disparities in risk stratification, diagnosis, and access to preventive interventions. Joint modeling of sex, gender, and ancestry is therefore not only a methodological refinement, but a prerequisite for equitable and clinically responsible implementation of AI-driven precision medicine in AD.

The second conceptual barrier, rooted in the “assuming differences” view, arises from gender stereotypes that prompt clinicians to interpret women’s symptoms through culturally shaped expectations rather than actual evidence. Research shows that ideas about femininity and masculinity (such as the belief that women are more emotional or men are braver) can skew diagnostic reasoning, steering it toward social assumptions instead of neutral clinical observation [97].

This stereotype-driven interpretative frame often leads to subtle “double standards”, meaning that the same behaviours or reported experiences are evaluated differently depending on the patient’s gender [98]. This becomes particularly relevant in many areas of the humanities, where research methods rely heavily on self-reporting, shaped by social norms. In fact, women and men learn to express distress, emotion, capability, or vulnerability in different ways [99], distorting how clinicians interpret what patients say and reinforcing biased assumptions instead of capturing their actual condition.

This bias is particularly evident in cognitive assessment. Studies show that women outperform men on verbal-memory tasks even in preclinical or early symptomatic AD, masking underlying pathology and delaying diagnosis [39,40]. In contrast, behavioural symptoms including apathy, disinhibition, and irritability are more readily pathologized in men, influencing staging, care pathways, and referral patterns [100]. These gendered interpretative discrepancies directly affect treatment access and clinical trial enrolment, reinforcing inequities [5,6].

Building on this, once differences are recognized at the population level, another risk emerges: clinicians may overgeneralize these findings to individual patients. Minor or irrelevant differences may be overstated or treated as purely biological. Once gender-related differences in a condition are described, they can end up driving gender-biased assessments of individual patients in practice. This process is known as “knowledge-mediated bias” [101].

As mentioned in the previous paragraph, differences in cognitive reserve, education, occupational complexity, and caregiving responsibilities contribute significantly to symptom onset [30]. Overemphasizing biological causation risks obscuring modifiable sociocultural determinants (such as chronic stress, sleep disruption, and unequal access to healthcare) that disproportionately affect women and may interact with neurobiological aging trajectories [102].

Clinically, sex- and gender-informed diagnostic pathways have the potential to improve early detection in women (who often present with higher neuropathological load despite preserved verbal performance) and refine prognostic stratification in men (who more frequently exhibit vascular/mixed pathology and behavioural symptoms [6]).

### Laboratory Diagnostics in AD: Integrating Sex and Gender for Enhanced Effectiveness

Biological sex influences multiple AD-related biomarkers, including amyloid-β isoforms, phosphorylated tau species, neurofilament light chain (NfL), glial fibrillary acidic protein (GFAP), lipidomic signatures, and neuroinflammatory markers, through both genetic and hormonal mechanisms.

Beyond biology, sociocultural gender factors (e.g., educational background, health-seeking behavior, caregiving responsibilities, and gendered diagnostic expectations) can alter diagnostic assessment. These gendered pathways influence not only biomarker concentrations but also their predictive value. Table 3 outlines key strategies for systematically incorporating sex and gender considerations to improve the accuracy and clinical utility of AD laboratory biomarkers.

From an operational perspective, biological sex should be encoded as a biological variable in experimental design and analytical models, either through stratified analyses or interaction terms with biomarkers and genetic risk factors. In contrast, gender should be operationalized through measurable sociocultural variables-such as education, occupational complexity, caregiving burden, socioeconomic status, and health-seeking behavior, rather than treated as a proxy for sex. In AI-based models, these dimensions should be represented as distinct features, enabling transparent evaluation of their independent and interactive contributions to risk prediction and biomarker interpretation.

Altogether, integrating both sex-specific biology and gendered life-course exposures into laboratory diagnostics enhances the accuracy, interpretability, and clinical utility of AD biomarker assessments. The rationale for incorporating sex and gender considerations into diagnostic technologies aligns with the gender impact assessment model described by Appendino et al. [103]. This dual-axis approach not only refines early detection and differential diagnosis but also supports personalized therapeutic strategies and equitable access to emerging disease-modifying treatments.

AI is increasingly being recognized for its potential in the prevention and treatment of dementia, alongside other complex, disabling or lethal diseases. However, numerous challenges and critical issues remain. At the same time, the role of AI in mitigating or exacerbating existing biases warrants critical consideration. AI holds promise for improving risk stratification and diagnostic accuracy in AD, including the potential to uncover sex- and gender-specific patterns that traditional analytical approaches may overlook. However, its role in addressing gender bias remains inherently ambivalent. AI systems trained on datasets that are unbalanced with respect to sex, gender, ancestry, or socioeconomic status risk perpetuating or amplifying existing diagnostic and structural biases. Without explicit incorporation of sex- and gender-aware design principles, algorithmic transparency, and bias auditing, AI-driven tools may reproduce the same inequities they aim to overcome.

Integrating biological evidence with gender-sensitive epidemiology and explicitly recognising structural gender biases is essential for advancing a precision-medicine framework that is both scientifically rigorous and socially equitable. Addressing conceptual biases alongside biological differences will enable the development of personalised interventions that more accurately reflect the true heterogeneity of AD and promote more effective and equitable care for all individuals at risk of, or living with AD. In parallel, emerging technological innovations are redefining the tools available for precision assessment and intervention. AI, in particular, is increasingly recognised for its potential to prevent, early detect, and optimize treatment of dementia, offering opportunities to enhance risk stratification, refine biomarker interpretation, and design more tailored, sex-responsive therapeutic pathways.

Taken together, these controversies highlight that innovation in AD prevention does not depend solely on technological advancement, but on the critical integration of biological validity, equity considerations, and clinical interpretability. Both PRS and AI-based tools should therefore be viewed as evolving components of a broader precision-medicine framework, whose responsible translation into practice requires rigorous validation, interdisciplinary oversight, and explicit attention to sex, gender, and population diversity.

This review has several limitations. First, its narrative design, while appropriate for integrating heterogeneous evidence across multiple disciplines, does not provide the methodological rigor of a systematic review and may be subject to selection bias. Nevertheless, this approach was chosen to enable conceptual integration across domains that are rarely addressed jointly and to capture emerging evidence not yet amenable to formal meta-analytic synthesis.

Future research should prioritize longitudinal and real-world studies to evaluate the long-term effectiveness, adherence, and equity impact of AI-personalized prevention strategies in AD. In particular, prospective cohorts and pragmatic trials will be essential to assess whether AI-guided, lifestyle-based interventions can dynamically modify risk trajectories over time and whether their benefits are consistent across sex, gender, and ancestry groups. Such evidence is critical to translate conceptual precision-medicine frameworks into clinically meaningful and equitable prevention strategies.

## 5. Conclusions

Understanding AD in 2030 will increasingly benefit from AI, with AI integrated across all aspects of prevention, risk assessment, diagnosis, and treatment. The rapid expansion of digital is impacting our daily activities. This transformation has revolutionized the healthcare field, thanks to the significant benefits it represents in terms of cost savings and patient management, even in the early stages and primary prevention. Currently, a wide range of proof-of-concept studies demonstrate the value of these digital tools for diagnosing, prognosing, and monitoring dementia, yet clinical practice remains largely unchanged. Large-scale studies and related big data processing pipelines are needed to formalize which measurement tools and devices are most promising for practical and clinical applications.

Genetic, sex, and gender differences in AD are pervasive and encompass risk, resilience, biological mechanisms, clinical expression, and response to therapy. Integrating sex- and gender-sensitive approaches into observational cohorts, genetic risk assessments, biomarker development, and interventional studies is a prerequisite for true precision medicine and prevention in AD.

This could improve the level of integration with AI tools to promote early diagnosis in women, refine risk stratification in men, and ultimately enable more effective and equitable prevention and treatment strategies for all individuals at risk of or living with AD. Regulatory approval pathways must be clarified and adapted, and greater flexibility is needed, as technology and data science evolve rapidly, with growing implications for personal and professional ethics.

Looking ahead, research at the intersection of genomics, sex/gender biology, and AI should focus on a limited number of concrete objectives over the next five years. First, large-scale, multi-ancestry genomic studies incorporating sex-stratified analyses are needed to improve the robustness and portability of PRS models and to enable equitable genetic risk prediction. Second, the systematic integration of sex- and gender-informed biomarkers across fluid, imaging, and digital domains should be prioritized to refine early diagnosis, risk stratification, and treatment monitoring. Third, the development and validation of AI models explicitly designed to jointly incorporate sex, gender-related exposures, and ancestry, with built-in transparency and bias auditing, will be essential to ensure clinical reliability and fairness. Finally, longitudinal and pragmatic studies are required to assess whether AI-personalized, lifestyle-based interventions can sustainably modify AD risk trajectories across diverse populations.

## Figures and Tables

**Table 1:** Key studies shaping the evolution of precision genomics and risk prediction in Alzheimer’s disease.

Study (First Author, Year)	Strategy	Biological Mechanisms Highlighted	Key Discoveries	Impact on AD Risk Prediction/Precision Genomics
Lambert et al., 2013 [11]	Large GWAS meta-analysis (International Genomics of Alzheimer’s Project, European ancestry)	Lipid metabolism, endocytosis, innate immunity	Identification of 11 novel loci and confirmation of APOE	Foundation for genome-wide PRS; expanded risk loci
Kunkle et al., 2019 [12]	International Genomics of Alzheimer’s Project -extended GWAS meta-analysis	Amyloid, tau, immunity, lipid processing, HLA region	Discovery of 5 additional loci and HLA-DR15 haplotype	Refined polygenic architecture and highlighted immune pathways
Bellenguez et al., 2022 [13]	Very large, multi-ancestry GWAS	Microglial activation, synaptic regulation, endosomal trafficking	42 new genome-wide loci, enriched in microglia/astrocytes	Highlighted ancestry effects and expanded PRS target variants
Zhang et al., 2020 [16]	PRS modelling and architecture evaluation	Lipid metabolism, endosomal processing, microglial activation	Oligogenic-like architecture; limited high-impact variants	Guided development of parsimonious, mechanism-focused PRS
O’Neill et al., 2025 [17]	Cell-type-specific PRS (multi-omic with snRNA/snATAC)	Microglia- and astrocyte-specific regulatory programs	Distinct ct-PRS linked to amyloid plaques, tau, cognitive decline	Enabled cell-type resolved polygenic risk stratification
Venkatesh et al., 2025 [18]	Integrative multi-omic PRS (genomic, transcriptomic, epigenomic)	Neuroinflammation, lipid metabolism, synaptic dysfunction	Multi-omic PRS outperforms traditional genome-wide PRS	Increased accuracy via functional variant prioritization
Qu et al., 2025 (BrainGeneBot) [19]	LLM/Transformer-informed variant prioritization	Immune–microglial, synaptic, lipid-processing networks	Improved biological interpretability without loss of accuracy	AI-driven PRS prioritization reflecting mechanistic relevance
Nicolas et al., 2025 [20]	Cross-ancestry PRS transferability evaluation	Ancestry-specific allele frequencies, linkage disequilibrium, regulatory architecture	European PRS underperform in non-European cohorts	Need for ancestry-aware PRS for equitable precision genomics
Wang et al., 2025 [22]	Multimodal diagnostic technologies	Imaging, fluid biomarkers, genomics, non-invasive detection	Integration improves early detection & classification	Positioned genomics within broader diagnostic precision frameworks
Arafah et al., 2023 [23]	Precision medicine clinical framework review	Genomics, multi-omics, biomarkers, targeted therapies	Framework for genomics + biomarkers + tailored therapy	Linked molecular stratification to personalized clinical intervention

Legend: AD, Alzheimer’s disease; GWAS, genome-wide association study; PRS, polygenic risk score; APOE, apolipoprotein E; HLA, human leukocyte antigen; snRNA-seq, single-nucleus RNA sequencing; snATAC-seq, single-nucleus assay for transposase-accessible chromatin sequencing; ct-PRS, cell-type-specific PRS; multi-omic, integration of multiple molecular layers (e.g., genomic, transcriptomic, epigenomic); LLM, large language model. Clinical note: clinical utility of PRS depends on ancestry-appropriate calibration and is expected to be highest when integrated with clinical and biomarker information for risk stratification, prevention/trial enrichment, and personalized monitoring.

**Table 2:** Sex and Gender differences in Alzheimer’s Disease and their interaction.

Sex-Based Biological Determinants of AD	Gendered Life-Course Exposures and Sociocultural Determinants	Interactions Between Sex Biology and Gendered Environments
Differences arising from genetically and physiologically determined factors, including genomic architecture, hormonal milieu, metabolic regulation, immune function, and neurobiological pathways.	Sociocultural determinants include gendered roles, life-course opportunities, cultural expectations, health behaviours, and differential access to healthcare services.	Emergent phenomena that result not from sex biology or gendered environments alone, but from their reciprocal interplay, which modulates disease risk, clinical progression, and pathological burden.
Estrogen-mediated neuroprotection; menopause-related vulnerability; hormone–immune interactions [27,28,29]	Global inequities in education, occupation, cardiovascular risk, and socioeconomic conditions [6,30].	Sex hormones modulate inflammatory and autophagic pathways interacting with environmental exposures [27,31]
More substantial APOE-ε4 effects in women; amplified tau phosphorylation; sex-specific transcriptomic dysregulation [29,32,33]	Caregiving burden, chronic stress, sleep disruption, depression; region-specific vascular and lifestyle exposures [6,34]	APOE-ε4 × sex effects shaping amyloid–tau processes under environmental modulation [29,35]
Heightened microglial activation; sex-dependent coupling of sTREM2/clusterin with tau and NfL [31,35]	Gendered health behaviours (smoking, alcohol use, physical inactivity) and unequal access to healthcare [6,34]	Immune and metabolic pathways influenced by gendered stress, lifestyle, and environmental adversity [34,36]
Sex-specific lipidomic vulnerability: reduced unsaturated lipids and omega-3 carriers in women; APOE–lipid interactions [37,38]	Gendered social roles, occupational patterns, and cumulative environmental exposures influence risk trajectories [30,34]	Lipid metabolism interacts with APOE genotype and lifestyle in a sex-dependent manner [37,38]
Cognitive phenotype differences: women show greater tau burden at similar amyloid levels [35,39]	Diagnostic bias due to verbal-memory advantage delaying women’s diagnosis; region-specific sociocultural determinants [6,40]	Sex-modulated biomarker trajectories (amyloid→tau and tau→cognition) under gendered environmental exposures [31,35]
Sex-dependent response to disease-modifying therapies; differential ARIA susceptibility [41,42]	Women are underrepresented in trials; there is a lack of sex-stratified analyses; global disparities in access to novel treatments [43,44]	Treatment efficacy and adverse events are shaped by sex, pathology and environmental interactions [43,45]
Non-pharmacological interventions show sex-specific neuroinflammatory and metabolic responses [36,46]	Gendered lifestyle determinants influencing adherence and exposure to protective behaviours [34,47]	Physical activity, diet, and vascular health exert sex-dependent effects on neuroinflammation and tau [36,47]

Legend: AD, Alzheimer’s disease; APOE, apolipoprotein E; tau, microtubule-associated protein tau; sTREM2, soluble triggering receptor expressed on myeloid cells 2; NfL, neurofilament light chain; ARIA, amyloid-related imaging abnormalities. Clinical note: Sex- and gender-informed interpretation may improve (i) risk assessment and counseling (including APOE-ε4-associated risk differences), (ii) biomarker interpretation and longitudinal monitoring, including consideration of menopausal status and related hormonal transitions (sex-modulated biomarker trajectories) and (iii) therapeutic decision-making and safety surveillance (e.g., ARIA risk and potential differential response to disease-modifying therapies). These considerations also motivate sex-balanced recruitment and routine sex-stratified analyses in clinical trials and real-world implementation.

**Table 3:** Strategies to Improve Laboratory Diagnostics through Sex and Gender Integration.

Strategic Area	Description	Sex/Gender Considerations
Sex-stratified reference ranges	Use biomarker cut-offs calibrated by sex, disease stage, and physiological context.	p-tau231, p-tau217, NfL, GFAP; menopausal status; APOE genotype.
Sex/biomarker interaction models	Incorporate interaction terms into ML models and diagnostic algorithms.	Sex/APOE-ε4; sex/tau; sex/NfL.
Pre-analytical stratification by sex and gender	Identify modifiers of biomarker stability and interpretability.	Hormonal status, menopause, sleep, depression, lifestyle, vascular risks.
Trial-ready sex/gender-aware frameworks	Apply sex and gender-informed criteria in therapeutic eligibility and safety monitoring.	ARIA susceptibility, biomarker-therapy coupling, differential progression rates.

Legend: ML, machine learning; APOE, apolipoprotein E; p-tau, phosphorylated tau at threonine; NfL, neurofilament light chain; GFAP, glial fibrillary acidic protein; ARIA, amyloid-related imaging abnormalities. Clinical note: Incorporating sex and gender can reduce systematic misclassification (via sex-calibrated thresholds), improve model performance and generalizability (via interaction-aware modelling), and enhance treatment safety/benefit assessment (e.g., ARIA risk monitoring and biomarker–therapy coupling), particularly around menopause-related physiological transitions and genotype-specific effects.

## Data Availability

No new data were created or analyzed in this study.

## Abbreviations

The following abbreviations are used in this manuscript:

AD	Alzheimer’s disease
AI	artificial intelligence
APOE	apolipoprotein E
APP	amyloid precursor protein
ARIA	amyloid-related imaging abnormalities
AUC	area under the curve
ct-PRS	cell-type-specific polygenic risk score
EU	European Union
FINGER	Finnish Geriatric Intervention Study to Prevent Cognitive Impairment and Disability
GDPR	general data protection regulation
GFAP	glial fibrillary acidic protein
GWAS	genome-wide association study
GRS	genetic risk score
HLA	human leukocyte antigen
LLM	large language model
LOAD	late-onset Alzheimer’s disease
MCI	mild cognitive impairment
ML	machine learning
NfL	neurofilament light chain
PD	Parkinson’s Disease
PET	positron emission tomography
PRISMA	Preferred Reporting Items for Systematic Reviews and Meta-Analyses
PRS	polygenic risk score
RCT	randomized controlled trial
SNP	single-nucleotide polymorphism
snATAC-seq	single-nucleus Assay for Transposase-Accessible Chromatin using sequencing
snRNA-seq	single-nucleus RNA sequencing
WHO	World Health Organization
